# Effect of different salt additions on the taste and flavor-related compounds in chicken soup

**DOI:** 10.3389/fnut.2024.1368789

**Published:** 2024-03-13

**Authors:** Rong Jia, Xiaoyan Yin, Yucai Yang, Guozhou Liao, Dahai Gu, Yuehong Pu, Guiying Wang

**Affiliations:** ^1^College of Food Science and Technology, Yunnan Agricultural University, Kunming, China; ^2^Livestock Product Processing and Engineering Technology Research Center of Yunnan Province, Yunnan Agricultural University, Kunming, China

**Keywords:** chicken soup, different salt additions, water-soluble small molecule compounds, free fatty acids, volatile compounds, LC-Q/TOF-MS, HS-SPME-GC–MS

## Abstract

Chicken soup is popular among consumers because of its delicious taste, strong flavor, and abundant nutritional value. Twenty-four Yunnan local hens were stewed by adding different amounts of NaCl [1.5, 2, 2.5, 3%, m/m, calculated based on chicken carcass weight; chicken: water = 1:2 (m/m)] to study the effect of salt addition on taste- and flavor-related compounds in chicken soup. Sensory evaluation results showed that the 2 and 2.5% NaCl treatment groups had higher scores. Water-soluble small molecule compounds were detected by LC-Q/TOF-MS based metabolomics approach, among which amino acids and their derivatives, nucleic acids, and small peptides were the main components. The concentration of Water-soluble small molecule substances in chicken soup samples with different salt additions showed a clear trend of separation and reached the highest in the 2.5% NaCl treatment group. Volatile flavor compounds in the chicken soup were analyzed by HS-SPME-GC–MS, including aldehydes, and alcohols, and the relative concentration of flavor compounds in the 2.5% salt treatment group was the highest. In summary, the addition of salt could improve the overall flavor of chicken broth, and the optimal salt addition of NaCl in chicken soup is 2.5%.

## Graphical abstract

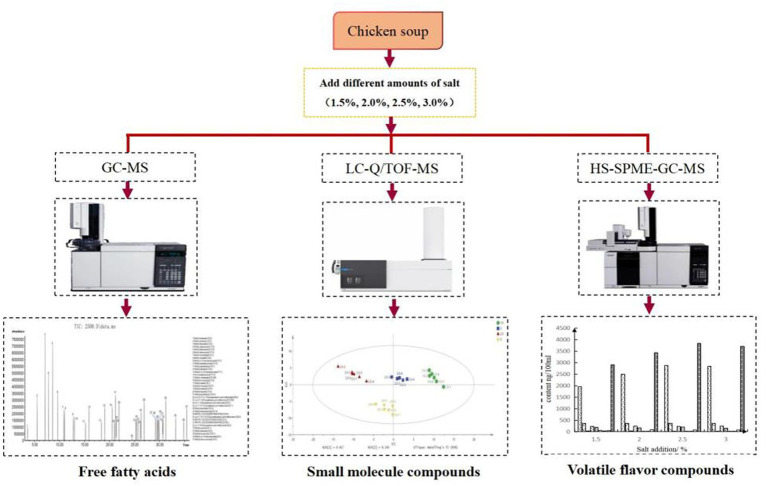

## Highlights

- Amino acids and their derivatives, and nucleic acids were the main components.
- Fifty free fatty acids were detected by GC-MS.
- Palmitic acid and stearic acid were the highest.
- Aldehydes were the main volatile compounds in chicken soup by HS-SPME-GC-MS.
- The optimal amount of salt addition was 2.5%.

## 1 Introduction

Chicken, the second-largest meat consumed in China, is considered a nutritional supplement because of its low fat, low cholesterol, low calorie, and high protein content (1). Yunnan Wuding chickens, famous for their tender meat and umami taste, are mostly distributed in cold mountainous areas, and often adopt the form of free range to get more minerals, insects, and other natural and good feed. Stewing is the most traditional cooking method for chicken (2). The nutritional components of chicken soup include protein, fat, vitamins, trace elements, and bioactive substances (3), which can enhance immunity, relieve cold symptoms, promote milk secretion, and improve anxiety (4), and they also play a physiological role in promoting metabolism, improving anemia, and enhancing antioxidant activity (5). With the deepening of people’s understanding of chicken, the potential development of the chicken market in the future will be huge.

Its high nutritional value and unique flavor have made chicken soup one of the most popular soups in China. Chicken soup contains a variety of taste and aroma substances, of which the taste substances mainly come from free amino acids, nucleotides, and other substances, and the aroma substances mainly come from aldehydes, alcohols, ketones, carboxylic acids, and sulfur heterocyclic volatile flavor substances (3). While cooking chicken, various water-soluble components are released into the soup, including peptides, nucleotides, soluble amino acids, carbohydrates, inosine, organic acids, and other substances. Li et al. found that amino acids influence the flavor of the chicken broth, and umami amino acids and their derivatives make the largest contribution to the taste of chicken broth (4). The 5′-inosine monosodium (IMP) and chloride ions are the main flavor components of chicken soup, which is conducive to the formation of chicken soup flavor components (6).

Salt can not only adjust the taste of chicken soup but also improve its overall quality (7). Salt has an effect on the oxidation of fat, the solubilization and denaturation of proteins, and the amount and method of addition have a significant impact on the quality of chicken soup (8). It can also activate proteins, increase their binding properties, improve their ability to bind with water, and affect some chemical and biochemical phenomena (7, 8). In terms of nutrient dissolution, stewed chicken soup with normal saline can increase the release of protein and minerals.

However, salt can also cause some diseases, such as high blood pressure (9). Excessive absorption of sodium ions in salt into human blood will lead to the retention of water sodium, which will lead to the increase of blood volume, and then make blood pressure rise (10). In addition to the amount of salt added, some processing technologies will also affect the quality of chicken soup, such as increasing the spillover substances and improving the nutritional components. Singh et al. (11) found that the amount of salt has the greatest impact on the flavor of chicken soup, and Sun et al. (12) demonstrated that the addition of different spices could harmonize the chicken soup and give it a unique flavor. Cooking utensils and processing conditions also have an effect on the quality of chicken soup, and ceramic cooking utensils produce more delicious results (13). Cooking time has a great influence on the concentration of spillage in chicken soup. Within a certain time range, the quality of chicken soup is positively correlated with cooking time (14).

In addition, the flavor characteristics of chicken soup are also related to chicken breeds. Due to the genetic differences among different chicken breeds and the different flavor substances deposited in chicken meat, there are great differences in the nutritional components and flavor substances of chicken soup. At present, there are few systematic studies on the effects of salt on the taste and aroma compounds of chicken soup. Therefore, in this study, hybrid F1 hens of Wuding chicken and Digao chicken were selected and braised with different amounts of salt, to explore the difference of flavor substances in chicken soup with the amount of salt and screen out the best amount of salt. The free fatty acids, volatile flavor compounds, and water-soluble small molecule compounds in the chicken soup were identified by gas chromatography–mass spectrometry (GC–MS), head space solid phase microextraction coupled with gas chromatography mass spectrometry (HS-SPME-GC–MS), and liquid chromatography-quadrupole/time of flight-mass spectrometry (LC-Q/TOF-MS) coupled with multivariate data analysis, respectively. LC-Q/TOF-MS has very high sensitivity and a very wide analysis range. HS-SPME-GC–MS can complete the separation, identification, and qualitative and quantitative analysis of complex mixed volatile components. It has the characteristics of short detection time, high efficiency, good accuracy, and solvent-free, and is a new green sample pretreatment technology. Furthermore, the overall flavor differences of chicken soups were evaluated by sensory evaluation. The results could provide a scientific and theoretical basis for the development and utilization of high-quality native chickens.

## 2 Materials and methods

### 2.1 Materials

A total of twenty-four 200-day-old crossbred F1 hens of Yunnan Wuding chicken and Digao chicken were randomly selected under the same breeding conditions, and collected at the same time in the Experimental Chicken Farm of Yunnan Agricultural University. The chickens were randomly divided into four groups, with six chickens in each group cooked separately, equivalent to a group of six parallel chickens. After slaughtering, the head, neck, and claws were removed, and then the carcasses were washed and collected for stewing. The weight of each carcass was about 1805 ± 85 g, and the weight of each leg was shown in Supplementary Table S1. The experimental procedure and protocol were approved by the Animal Care Committee of the College of Animal Science and Technology, Yunnan Agricultural University.

### 2.2 Sample preparation

According to the traditional chicken soup stewing process, the chicken carcass was boiled in boiling water for 3 min, then washed with cold water, drained, and weighed. The chicken and ultrapure water were put into the casserole (Andy, China) according to the ratio of chicken: water = 1:2 (m/m) and put on the induction cooker (Midea, China) to boil at 2100 W. The scum on the upper layer was removed, and varying amounts of 1.5, 2, 2.5, and 3% salt (calculated based on chicken carcass weight, the salt purity of 99.9%, from the local supermarket) were added to the four treatment groups, respectively. The chicken was then stewed over low heat (300 W) for 2.5 h and started counting with water boiling. After stewing, enough warm ultrapure water was added to restore the original weight of the chicken soup. And 30 mL of chicken soup samples were collected in brown bottles for chemical index analysis. Meanwhile, the remaining chicken soup samples were collected for sensory evaluation.

### 2.3 Sensory evaluation

The sensory evaluation method for chicken soup was modified slightly from our previous method (15). The chicken soup was placed in a clean, disposable cup, and 16 graduate students majoring in food science were invited to conduct sensory evaluation, with a male-to-female ratio of 1:1. Sensory panelists had some experience in sensory evaluation for at least 1 year, and they were all healthy, non-smoking panelists, and all panelists had no taste or smell impairments. The total score was calculated according to the corresponding weights of the four evaluation criteria, namely, X = 0.15×_1_+ 0.4×_2_+ 0.3×_3_+ 0.15×_4_, where X_1_, X_2_, X_3_, and X_4_ represent the proportion of each weight and the specific sensory evaluation criteria were shown in Supplementary Table S2, which is mainly based on our research group previous research (2).

### 2.4 Free fatty acids analysis

The method used to determine the free fatty acids composition of chicken soup was according to our previous procedure (16). In a headspace bottle, 200 μL of the sample was placed, 3 mL of GC-grade n-hexane was added, vortexed for 1 min, and then centrifuged at 3500 rpm at 4°C for 5 min. A 60 μL pretreated sample was taken for detection. The FFA composition was determined by the 7890A-5975C GC–MS instrument (Agilent, Palo Alto, USA). The temperature of the FID inlet of the detector was 250°C, the flow rate of the injection volume was 0.3 mL/min, and the injection was 1 μL. Heating procedure: Started at 55°C, heated up at 30°C/min to 205°C, kept for 5 min, then heated up at 5°C/min to 230°C, and kept for 5 min, the total process was 40 min.

### 2.5 Water-soluble small molecule compounds analysis

The water-soluble small molecule compounds in chicken soup were measured using the method described in our previous report (15). A 100 μL sample was absorbed by a liquid-shift gun in a 1.5 mL EP tube; 800 μL methanol was added; and 10 μL internal standard (3 mg/mL of 2-chlorophenyl alanine) was added. The mixture was vortexed for 30 s and then centrifuged at 12000 rpm for 15 min, and 200 μL of the supernatant was absorbed and transferred to the injection bottle for detection. And 4 μL of the sample were loaded onto the separation column (DB-wax, Agilent, USA C18, 100 mm × 2.1 mm, 1.8 μm) and separated using solvent B with a 15 min linear gradient of 5–95% at a speed of 0.35 mL/min at 40°C (A: aqueous 0.1% formic acid, B: 100% acetonitrile/0.1% formic acid). The mass spectrometry data were retrieved from the METLIN and HMDB databases for qualitative analysis, and the relative peak area of the chromatogram was used for quantitative analysis.

### 2.6 Analysis of volatile compounds

The volatile compounds in the chicken soup were determined according to our previous study with minor modifications (8). A 5 mL of chicken soup samples were taken, the temperature was set at 60°C, the shaking speed was 250 rpm for 15 min, the extraction time was 30 min, the analytical time was 4 min, the GC cycle time was 57 min, and the internal standard was 200 ng (100 μg/mL × 2 μL) of 2-methyl-3-heptanone. The sample was separated by GC–MS (7890A-5975C, Agilent, Palo Alto, USA). The injection volume and temperature were 5 mL and 260°C, respectively, and helium was the carrier gas at a flow rate of 1 mL/min. The column temperature was maintained for 5 min at 40°C and for 5 min when the temperature rose to 250°C at 5°C/min. The temperatures of the ion source and quadrupole were 230°C and 150°C, respectively, and the scanning mode was full scanning in the mass range of 20–400 amu. Mass spectrometry libraries (Wiley7n and NIST2011) were used for qualitative analysis.

### 2.7 Statistical analysis

In accordance with the design principle of random grouping, six parallel experiments were designed for each group, with data expressed as mean ± standard deviation. Excel 2010 and SPSS 19.0 software were used to analyze the data (15). Duncan’s multiple range method was used to examine the multiple significant differences (*p* < 0.05). The principal component analysis (PCA), and partial least squares discriminant analysis (PLS-DA) were used for statistical analysis with SIMCA 14.1.

## 3 Results

### 3.1 Sensory quality

**Figure 1:**
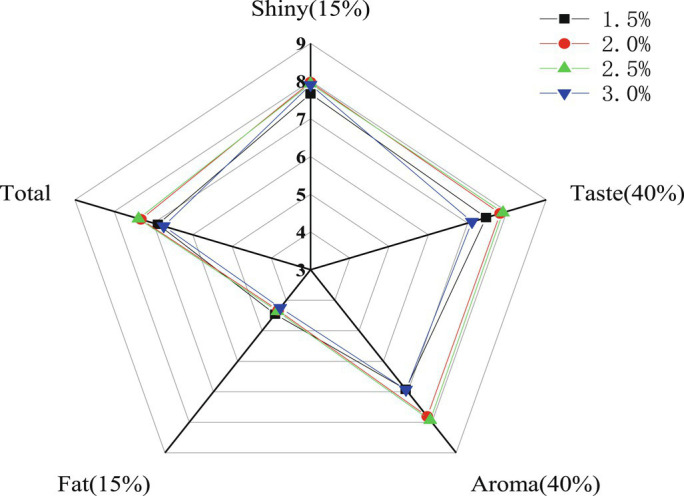
Sensory evaluation score of chicken soup in different treatment groups.

As one of the most commonly used additives in the food industry, salt plays an important role in the processing, preservation, and sensory acceptability of meat, and soups and has a great effect on the human senses (17). It can be seen from Figure 1 and Supplementary Tables S2, S3 that different salt additions had significant effects on the sensory quality of chicken soup (*p* < 0.05). When the salt addition was 2 and 2.5%, the total sensory scores of the chicken soup were the highest. Compared with other treatment groups, the chicken soup added 1.5% salt scored lower in terms of overall flavor and taste, which may be related to the lack of sensory synergy of salt on the taste components of chicken soup. The overall flavor and taste score of chicken soup with 3% salt was lower, which was possibly due to excessive salt. The results showed that neither low nor high salt concentrations had good sensory effects. Relative to protein nutrition and sensory acceptance, 2.5% salt is the most appropriate.

### 3.2 Free fatty acids compositions

As can be seen from Table 1, a total of 15 free fatty acids were detected in chicken soup, of which saturated fatty acids accounted for the main component, about 5 times that of unsaturated fatty acids. The concentration of other fatty acids changed, but most of them were not significant, and the fatty acids with the highest concentrations were palmitic acid (C16:0) and stearic acid (C18:0) (6). The total fatty acid concentration ranged from 885.62 μg/ mL in the 1.5% salt group to 945.08 μg/ mL in the 2.5% salt group, which was significantly different in the four groups of decoctions (*p* < 0.05). In the range of 1.5 to 3% salt content, there were significant differences in C22:2 between 2.5 and 3% salt content groups and C14:0 between 1.5 and 2.5% salt content groups. The difference in saturated fatty acids, monounsaturated fatty acids, and polyunsaturated fatty acids concentration among the four groups of chicken soup was not significant (*p* > 0.05). The automatic oxidation of unsaturated fatty acids will produce hydrogen peroxide, which can continue to react to produce alcohols, ketones, aldehydes, and other flavor substances, which have an important influence on the flavor of chicken soup.

**Table 1:** Composition of free fatty acids in chicken soup of different treatment groups (μg/mL).

Free fatty acids	Different salt additions /%			
	1.5	2.0	2.5	3.0
C10:0	2.03 ± 0.01^a^	2.03 ± 0.04^a^	2.18 ± 0.23^a^	2.19 ± 0.13^a^
C12:0	3.74 ± 0.94^a^	3.81 ± 0.19^a^	4.05 ± 0.06^a^	3.99 ± 0.23^a^
C14:0	9.68 ± 1.37^a^	10.37 ± 0.42^ab^	10.65 ± 0.85^b^	10.95 ± 1.30^ab^
C14:1n5	3.30 ± 0.04^a^	3.45 ± 0.20^a^	3.66 ± 0.17^a^	3.51 ± 0.08^a^
C15:0	2.75 ± 0.09^a^	2.79 ± 0.09^a^	3.09 ± 0.26^a^	2.80 ± 0.22^a^
C16:0	497.16 ± 13.22^a^	528.02 ± 14.68^a^	548.76 ± 67.04^a^	529.55 ± 4.00^a^
C17:0	5.56 ± 0.56^a^	5.53 ± 0.13^a^	5.56 ± 0.60^a^	5.55 ± 0.29^a^
C18:0	361.08 ± 18.52^a^	361.29 ± 18.25^a^	367.23 ± 34.3^a^	360.32 ± 25.87^a^
C18:1n9c	19.56 ± 0.55^a^	22.32 ± 2.21^a^	22.62 ± 2.70^a^	20.05 ± 1.58^a^
C18:2n6c	77.30 ± 1.25^a^	77.99 ± 0.75^a^	78.81 ± 4.20^a^	78.19 ± 2.67^a^
C20:0	1.98 ± 0.08^a^	1.93 ± 0.04^a^	1.86 ± 0.30^a^	1.89 ± 0.35^a^
C20:1	1.41 ± 0.07^a^	1.48 ± 0.17^a^	1.54 ± 0.12^a^	1.43 ± 0.18^a^
C22:1n9	44.49 ± 1.88^a^	47.09 ± 2.30^a^	46.7 ± 2.32^a^	45.58 ± 1.48^a^
C22:2	7.69 ± 0.22^ab^	7.70 ± 0.05^ab^	7.86 ± 0.23^b^	7.26 ± 0.36^a^
C24:0	1.64 ± 0.06^a^	1.69 ± 0.25^a^	1.70 ± 0.06^a^	1.70 ± 0.20^a^
TF	1039.36 ± 36.23^a^	1077.49 ± 31.59^a^	1106.26 ± 93.28^a^	1074.93 ± 30.69^a^
SFA	885.62 ± 34.14^a^	917.46 ± 29.2^a^	945.08 ± 100.26^a^	918.92 ± 27.46^a^
MUFA	68.76 ± 1.90^a^	74.34 ± 3.41^a^	74.51 ± 5.18^a^	70.57 ± 2.82^a^
PUFA	84.99 ± 1.24^a^	85.69 ± 0.76^a^	86.67 ± 4.42^a^	85.44 ± 2.92^a^

Different letters in the same row indicate significant differences (*p* < 0.05).

Free fatty acids have significant antioxidant effects (18). The high level of saturated fatty acids in the diet is related to coronary heart disease and atherosclerosis. The high level of unsaturated fatty acids in the diet will increase the level of high-density lipoprotein cholesterol and reduce the levels of low-density lipoprotein cholesterol and triglycerides (19). Therefore, it is necessary to replace saturated fatty acids with unsaturated fatty acids in the diet. The four groups of chicken soups in this study were rich in unsaturated fatty acids, which meet the requirements of healthy food. With the increase in salt intake, most fatty acids in chicken soup increased first and then decreased, which may be a high concentration of salt that has an inhibitory effect on some fatty acids.

Different tastes may be due to differences in palmitic acid concentration in four groups of chicken soup, and palmitic acid and stearic acid have been found to be great sources of bioactive lipids and are needed for human development (20). According to research, medium- and long-chain free fatty acids (C > 6) can be degraded as substrates to create volatile taste chemicals such as aldehydes and acids (21). Different fatty acid compositions result in a variety of meat flavors. For example, the main fatty acids in cured duck meat are palmitic acid and stearic acid. The main fatty acids in pork are palmitic acid, stearic acid, oleic acid, and linolenic acid (22), but the lowest concentration of fatty acids in dry-cured ham is palmitic acid (23), while the most abundant free fatty acids in dairy products such as milk are 7-hydroxystearic acid and 10-hydroxystearic acid (24).

### 3.3 Water-soluble small molecule compounds

The (ESI+) and (ESI−) sample ion flow chromatograms of four chicken soup groups showed different salt additions are shown in Supplementary Figure S1 and Supplementary Table S4. TICs of samples were determined by chromatographic column, and mass spectrometry data were extracted and pretreated. One hundred thirty-five compounds were identified by liquid chromatography-mass spectrometry in four groups of decoctions, mainly including amino acids and their derivatives, vitamins, nucleotides, polypeptides, and organic acids. Although some differences can be observed in the sample ion flow chromatogram, many other visual changes can be observed by using pattern recognition methods, such as principal component analysis and partial least squares discriminant analysis. Principal component analysis was used to analyze the data obtained by liquid chromatography-mass spectrometry in positive and negative ion modes and different sample groups were separated by the partial least squares discriminant analysis method to further improve the recognition rate (16, 25).

**Figure 2:**
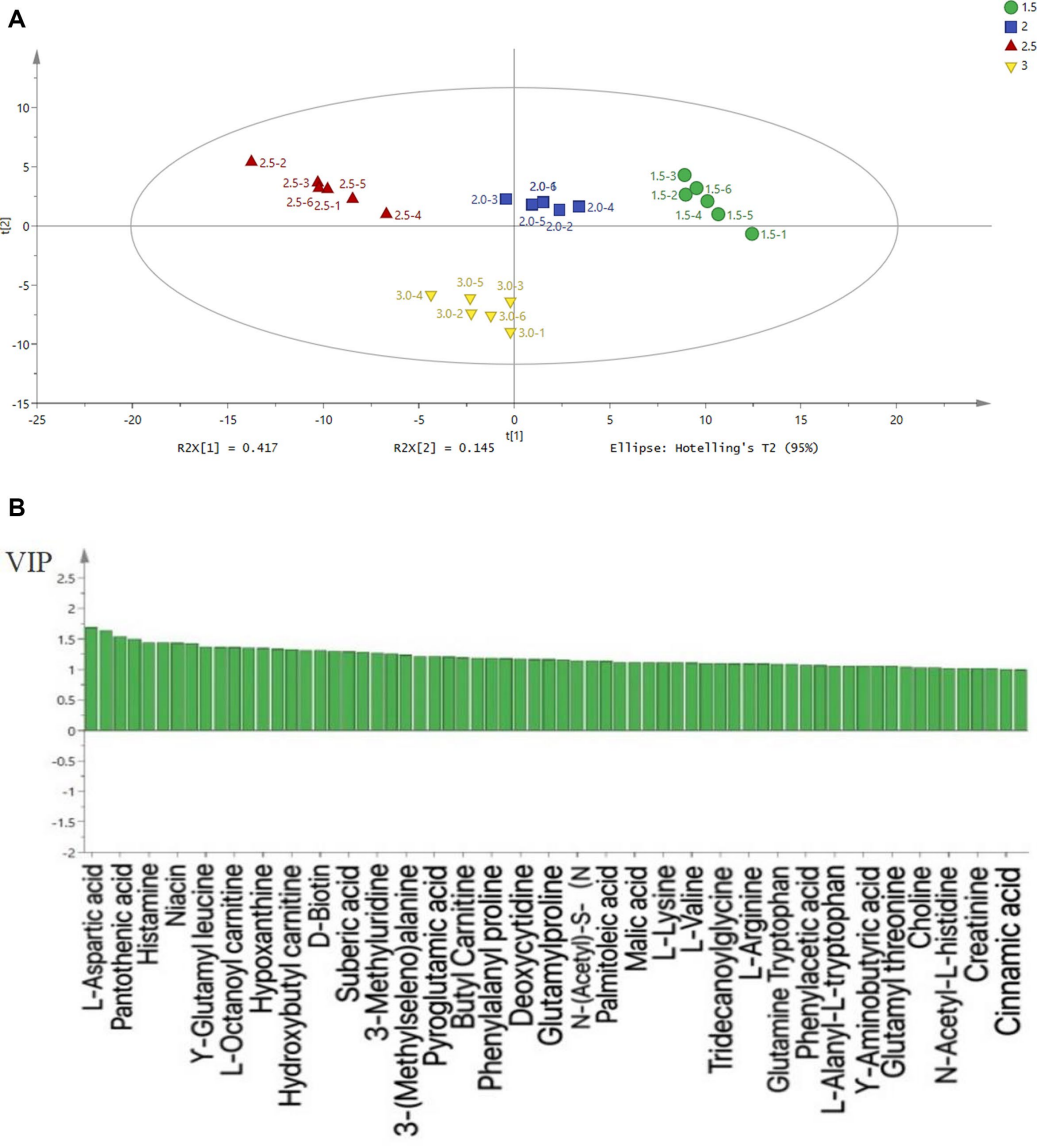
PLS-DA analysis **(A)** and VIP **(B)** results of water-soluble small molecule compounds in chicken soup. 1.5, 2, 2.5, and 3 in the figure represent the chicken soup treatment group with different salt addition amounts, and the salt addition amounts are 1.5, 2, 2.5, and 3%, respectively.

The principal component analysis of water-soluble small molecule compounds in chicken soup is shown in Figure 2, which is based on the statistical analysis results of 135 water-soluble compounds in chicken soup samples with different salt additions. R^2^Y = 0.626 and *Q*^2^ = 0.584 for PLS-DA score (a) pattern cross-validation, which can explain and predict the model’s ability. All samples were within the 95% confidence interval and had an obvious separation trend, indicating that principal component analysis was suitable for the analysis of water-soluble small molecule compounds in four groups of chicken soups with different salt additions. It can be seen from Figure 2 that the samples with different salt additions had a clear separation trend, indicating that the profiles of water-soluble metabolites are different.

As shown in Figure 2B, the concentrations of amino acids and their derivatives in four soups were the highest (26). According to their taste characteristics, amino acids can be divided into sweet, sour, bitter, salty, umami, and tasteless amino acids. Among them, umami amino acids and their derivatives contribute the most to the flavor of chicken soup (4). Glutamic acid and aspartic acid are considered to be the main delicious amino acids, and their umaminess-increasing effects are significant. In the process of stewing, different osmotic pressures have a certain influence on the dissolution of chicken, and the mutual transformation of flavor substances also affects the water-soluble taste in the chicken soup. From the VIP diagram (2b), it can be seen that the water-soluble substances of chicken soup samples with different salt additions are mainly aspartic acid and pantothenic acid. Amino acids and their derivatives, nucleic acids, polypeptides, and organic acids were the main components of water-soluble substances (26).

Chicken soup is a good source of essential amino acids, such as lysine, tryptophan, phenylalanine, isoleucine, leucine, and valine. The 5′-adenosine nucleotide and 5′-inosine hypoxanthine detected in four groups of soups may enhance the flavor of chicken soup (27). In addition to amino acids and 5′-nucleotides, other water-soluble small molecule compounds such as vitamins and peptides also have certain effects on the formation of the final flavor of chicken soup (26). In chicken, cysteine, methionine, lysine, myristic acid, palmitic acid, and stearic acid have a positive impact on fat properties, while glutamic acid, threonine, tyrosine, and isoleucine have a strong positive impact on umami properties and have been proven to be the main contributors to the flavor of chicken soup (28).

Some studies have also shown that nucleotides and some free amino acids, such as aspartate and glutamic acid are the main contributors to the delicious taste of chicken (4). Pantothenic acid, also known as vitamin B5, is a water-soluble vitamin necessary to maintain life that can enhance human resistance and play an important role in carbohydrate, fatty acid, protein, and energy metabolism (29). Histamine is an indispensable substance in the human body (30). The concentration of glutamic acid, glycine, histidine, and tyrosine in the soup increased significantly after the addition of salt, indicating that the protein in the soup is expected to be better absorbed and utilized in the human body. The flavor of chicken soup was mainly derived from nucleotides, amino acids, and their derivatives, and other water-soluble substances, which contributed a lot to the taste of chicken soup. When the addition of salt was 2.5%, the water-soluble flavor components were mainly pantothenic acid, nicotinic acid, and taurocholic acid.

### 3.4 Volatile flavor compounds

**Figure 3:**
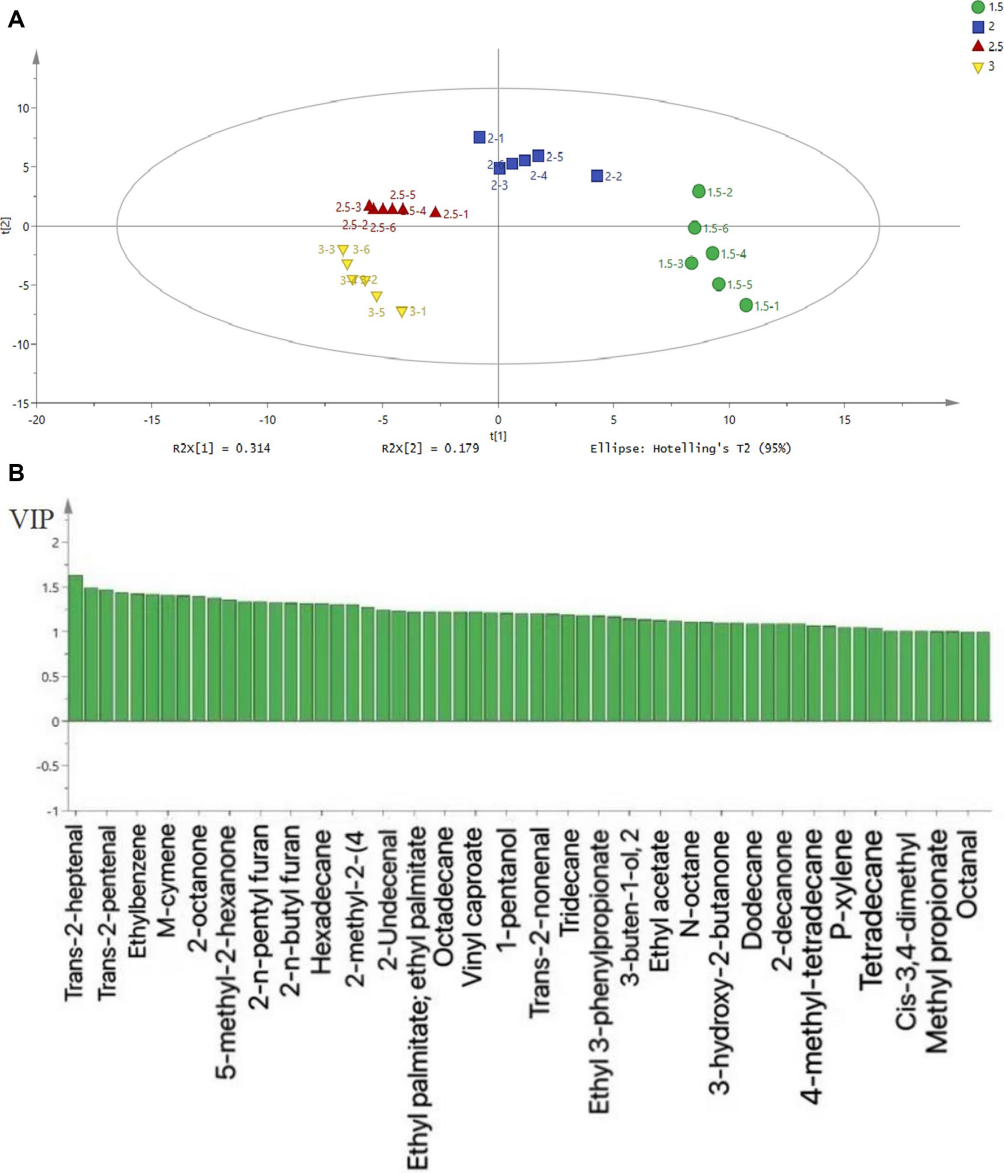
PLS-DA analysis **(A)** and VIP **(B)** results of volatile flavor components in chicken soup.

**Figure 4:**
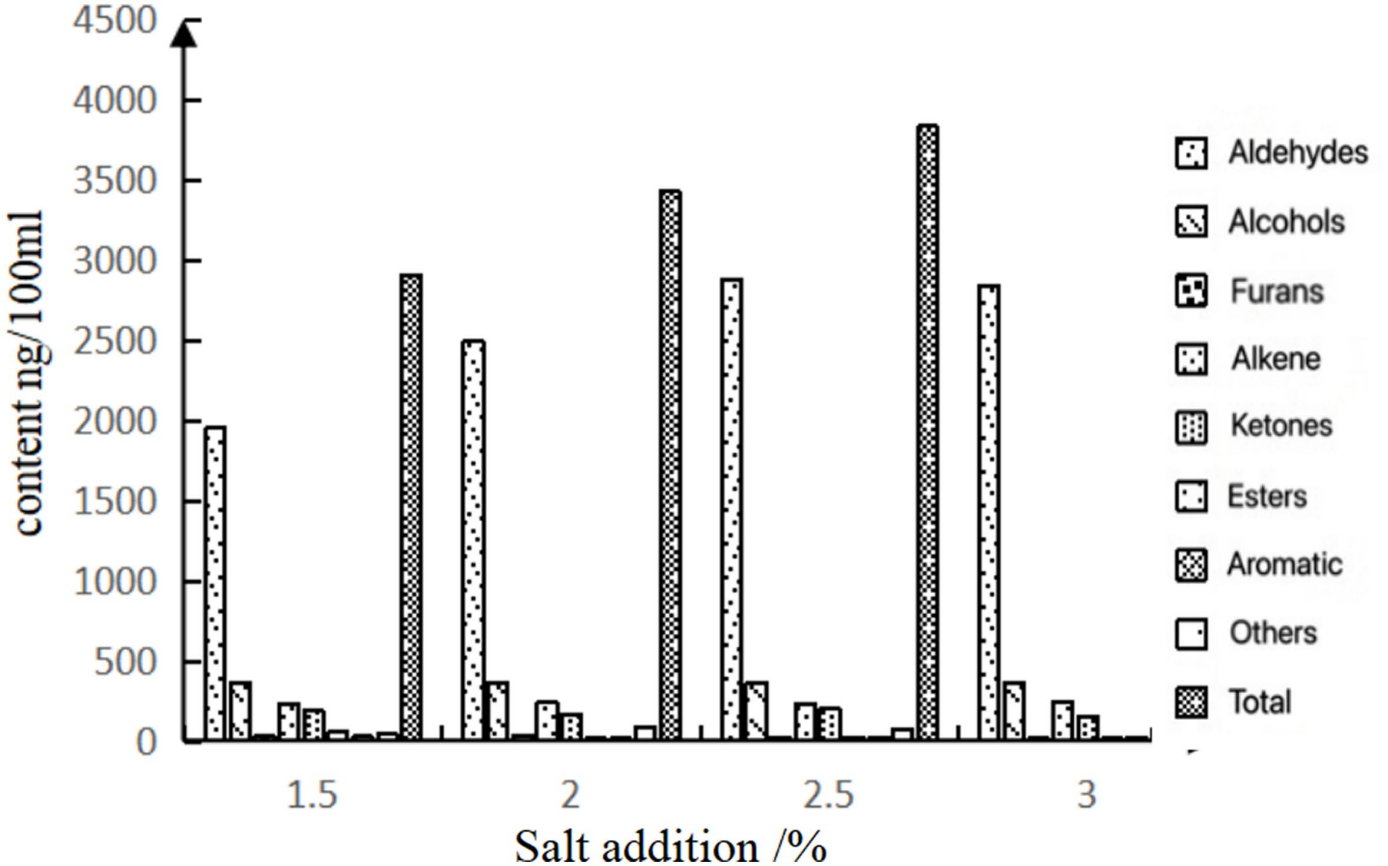
Concentration of volatile components in chicken soup of different treatment groups.

Volatile flavor compounds are the main factors affecting the formation of meat-specific flavors (8). The volatile compounds detected in the chicken soup were mainly aldehydes, alcohols, furans, alkanes, ketones, and aromatic compounds, which can be seen in Figures 3, 4 and Supplementary Table S5. The concentrations of aldehydes and alcohols in chicken soup increased first and then decreased with the increase in salt addition. The reason for this phenomenon may be that the addition of salt increases the osmotic pressure of chicken broth, promotes the transfer of aldehydes and alcohols, and is conducive to the formation of complex volatile flavor substances. However, with the increase of salt content in chicken soup, the osmotic pressure balance is destroyed, causing them to flow back to the interior of the chicken, or it may be due to the natural decomposing of some of them. From the perspective of volatile matter concentration, the concentration of salt in the 2, 2.5, and 3% groups was higher, and the concentration was the highest in the 2.5% group. The aldehydes in the 1.5% salt addition group were significantly different from other salt addition groups (*p* < 0.05). There was a significant difference in aldehydes between the 2% salt addition group and other salt addition groups, but there was no significant difference between the 2.5% salt addition group and the 3% salt addition group. Alcohol concentration changed with salt content, but there was no significant difference among the groups. It can be seen from Figure 4 that the contents of volatile compounds in the four groups of chicken soups were significantly different. Compared with other experimental groups, the difference gradually increased with the increase in gradient when the salt content was 1.5%. The addition of 2 and 2.5% salt had little difference, which was similar to the sensory evaluation.

Reasonable thermal oxidation changes of lipids may lead to satisfactory aromatic compounds in cooked meat (12), and the oxidation of fatty acid components will produce hundreds of volatiles, including aldehydes, ketones, alcohols, esters, and others, which are considered to be aroma compounds in chicken (31). The main aldehydes and volatile flavor compounds in four groups of chicken soup were hexanal, heptanal, octanal, nonanal, and decanal. The key flavor-related compounds in chicken soup are hexanal, heptanal, octanal, nonanal, 1-hexanol, and 2-pentylfuran (32). Studies have shown that aldehydes are the main volatile compounds in chicken soup, and allenal and dienal are considered to be the characteristic volatile components of chicken soup (13). Among the carbonyl compounds (penta, penta) -2, 4-decadienal, and (penta) -2-decenal are the most important components for the formation of chicken flavor (4), and olefin compounds are mainly derived from fat oxidation or amino acid oxidation; hexanal, trans-2-decenal, and 2-pentylfuran give chicken soup rich fat and meat flavor (30).

The addition of salt not only affected the dissolution of water-soluble substances but also had a great influence on the sensory evaluation of flavor components in chicken soup. One hundred thirty-five water-soluble substances and 15 free fatty acids were detected. When the addition of salt was 2.5%, the sensory evaluation of stewed chicken soup was the best, and the concentration of water-soluble substances was the highest. One hundred thirty-four volatile flavor compounds were detected in chicken soup, and aldehydes were the main flavor contributors. In conclusion, the chicken soup had the best sensory qualities, overall tastes, and flavors when the salt content was 2.5%. These results offered rational theoretical guidance for the deep processing of local chickens, and the next step could further explore the molecular mechanism of chicken soup with different salt additions that form unique flavors during processing.

## 4 Conclusion

The addition of salt not only affected the dissolution of water-soluble substances but also had a great influence on the sensory evaluation of flavor components in chicken soup. One hundred thirty-five water-soluble substances and 15 free fatty acids were detected. When the addition of salt was 2.5%, the sensory evaluation of stewed chicken soup was the best. At the same time, the concentration of water-soluble substances and free fatty acids in the chicken soup was the highest, and the water-soluble flavor components were mainly pantothenic acid, nicotinic acid, and taurocholic acid. The flavor of chicken soup was mainly derived from nucleotides, amino acids, and their derivatives, and other water-soluble substances, which contributed a lot to the taste of chicken soup. One hundred thirty-four volatile flavor compounds were detected in chicken soup, and aldehydes, alcohols, furans, and alkanes were the main flavor contributors. In conclusion, the chicken soup had the best sensory qualities, overall tastes, and flavors when the salt content was 2.5%. These results offered rational theoretical guidance for the deep processing of local chickens, and the next step could further explore the molecular mechanism of chicken soup with different salt additions that form unique flavors during processing.

## Data availability statement

The original contributions presented in the study are included in the article/Supplementary material, further inquiries can be directed to the corresponding authors.

## Author contributions

RJ: Conceptualization, Data curation, Formal analysis, Investigation, Methodology, Software, Validation, Writing – original draft, Writing – review & editing. XY: Conceptualization, Methodology, Validation, Writing – original draft, Writing – review & editing, Formal analysis, Investigation, Software. YY: Conceptualization, Formal analysis, Investigation, Methodology, Software, Validation, Writing – original draft, Writing – review & editing, Data curation. GL: Conceptualization, Data curation, Methodology, Validation, Writing – original draft, Writing – review & editing. DG: Data curation, Investigation, Methodology, Software, Writing – review & editing. YP: Investigation, Software, Writing – review & editing. GW: Formal analysis, Methodology, Writing – review & editing.
