# Supplementary material for: Effect of different salt additions on the taste and flavor-related compounds in chicken soup
Source: Front Nutr. 2024 Mar 13;11:1368789. doi: 10.3389/fnut.2024.1368789 (PMC10965538; doi:10.3389/fnut.2024.1368789)
Supplement: Supplementary file 1 [file Data_Sheet_1.doc]

ESI-

ESI+

**1.5%**

ESI-

ESI+

**2.0%**

ESI-

ESI+

**2.5%**

ESI-

ESI+

**3.0%**

**Fig. 1S** The representative total ion chromatograms of WSSW compounds in different salt addition groups detected by LC-MS in the positive（ESI+）and negative（ESI-）ion mode.


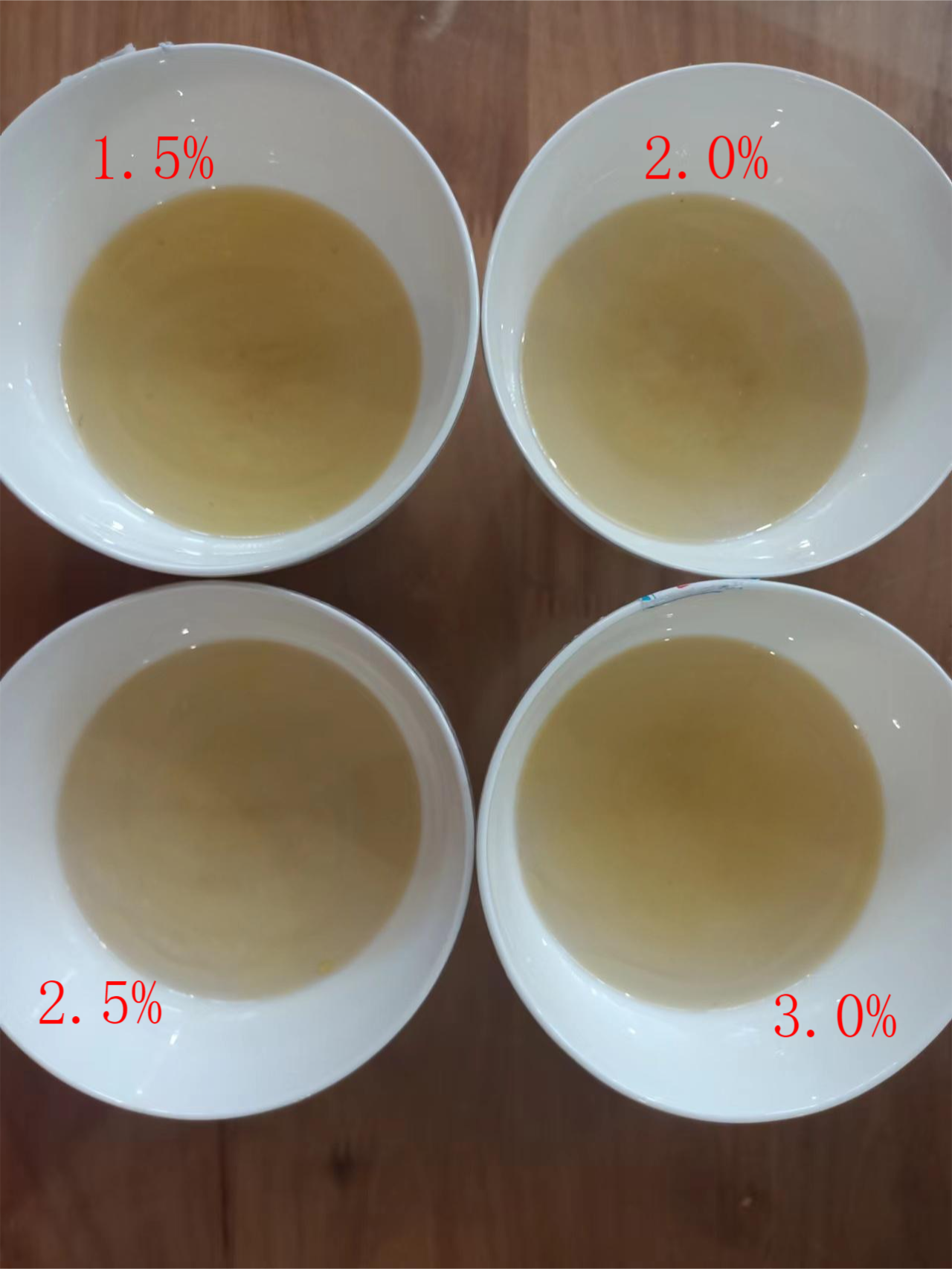


**Fig. 1S** Four groups of chicken soup samples.

Table 1S Carcass Weight of 24 chickens (200 days of age)

| Number | Different salt additions/% | Carcass Weight (g) | Number | Different salt additions/% | Carcass Weight (g) |
| --- | --- | --- | --- | --- | --- |
| 1 | 1.5 | 1880 | 13 | 2.5 | 1620 |
| 2 |  | 1835 | 14 |  | 1904 |
| 3 |  | 1798 | 15 |  | 1797 |
| 4 |  | 1785 | 16 |  | 1787 |
| 5 |  | 1807 | 17 |  | 1800 |
| 6 |  | 1907 | 18 |  | 1788 |
| 7 | 2 | 1900 | 19 | 3 | 1804 |
| 8 |  | 1703 | 20 |  | 1654 |
| 9 |  | 1690 | 21 |  | 1902 |
| 10 |  | 1990 | 22 |  | 1780 |
| 11 |  | 1850 | 23 |  | 1798 |
| 12 |  | 1756 | 24 |  | 1790 |

Table 2S Criteria for sensory evaluation of chicken soup

| Evaluation criteria | Score | | | |
| --- | --- | --- | --- | --- |
| Standard/ (weight) | 9~10 | 6~8 | 3~5 | 0~2 |
| Shiny（15%） | Pale yellow or milky white | beige | Light yellow | colorless |
| Taste（40%） | rich with a clear umami | Lack of umami, pure taste | Taste light, no aftertaste, no special odor | no umami, bad smell |
| Aroma（30%） | fragrant and rich meat smell | strong meat flavor, light aroma | Less meat smell, no peculiar smell | No meat smell, bad smell |
| Fat（15%） | no obvious oil slick on the surface of the soup | a small amount of particle precipitation | a lot of grease on the surface of the soup | The surface is covered with oil, and the grease layer is thicker |

Table 3S Result obtained for sensory evaluation of different treatment groups of chicken soup

| Evaluation criteria | Different salt additions/% | | | |
| --- | --- | --- | --- | --- |
| Standard/weight | 1.5 | 2.0 | 2.5 | 3.0 |
| Shiny（10%） | 7.66±0.02^a^ | 7.97±0.01^c^ | 7.93±0.03^b^ | 7.9±0.02^b^ |
| Taste（40%） | 7.47±0.01^b^ | 7.83±0.02^c^ | 7.90±0.09^c^ | 7.11±0.27^a^ |
| Aroma（30%） | 6.92±0.03^a^ | 7.81±0.02^c^ | 7.93±0.04^b^ | 6.93±0.06^a^ |
| Fat（10%） | 4.46±0.02^c^ | 4.33±0.02^b^ | 4.36±0.05^b^ | 4.25±0.05^a^ |
| Total | 6.89±0.01^b^ | 7.32±0.02^c^ | 7.38±0.03^c^ | 6.74±0.10^a^ |

Note: *P*<0.05 is represented between different lowercase letters of the same pair, and the same letter represents *P*>0.05.

Table 4S List of water-soluble compounds in chicken soup samples of each treatment group

| Metabolite | Metabolite | Metabolite |
| --- | --- | --- |
| L-Arginine | Thymidine 5'-triphosphate | Dodecanedioic acid |
| L-Arginine | Thymidine 5'-triphosphate | Dodecanedioic acid |
| L-Glutamate | Adenine | Fumaric acid |
| L-Histidine | Guanine | Oxoglutaric acid |
| L-Isoleucine | Thymine | Pimelic acid |
| L-Lysine | Uracil | Sebacic acid |
| L-Methionine | Alanylproline | Suberic acid |
| L-Norleucine | Alanyltryptophan | Terephthalic acid |
| L-Phenylalanine | gamma-Glutamylleucine | Arachidonic acid |
| L-Proline | Glutaminyltryptophan | Linoleic acid |
| L-Threonine | Glutaminylvaline | Oleic acid |
| L-Tryptophan | Glutamylphenylalanine | Palmitoleic acid |
| L-Valine | Glutamylproline | α-Linolenic acid |
| N-Acetylhistidine | Glutamylthreonine | Tetracosahexaenoic acid |
| N-Acetyl-L-methionine | L-beta-aspartyl-L-leucine | Hexadecanedioic acid |
| N-Acetylornithine | Leucyl-phenylalanine | Niacin |
| N-lactoyl-Methionine | Leucylproline | Niacinamide |
| N-Lactoylphenylalanine | L-phenylalanyl-L-hydroxyproline | Pantothenic acid |
| Pyroglutamic acid | L-prolyl-L-proline | Pyridoxamine |
| β-Alanine | Phenylacetylglycine | Riboflavin |
| γ-Aminobutryic acid | Phenylalanylproline | alpha-Tocopherol |
| 3-Methylhistidine | Prenyl-L-cysteine | Biotin |
| Betaine | Valylproline | 4-Pyridoxic acid |
| Creatinine | Anserine | B2Thromboxane B2 |
| N-Acetyl-S-(N-methylcarbamoyl)cysteine | Aspartylphenylalanine | Choline |
| Selenohomocysteine | Cysteinylglycine | Taurocholic acid |
| Taurine | Glutamylalanine | 9-Decenoylcarnitine |
| Tridecanoylglycine | Glutamylglutamic acid | Acetylcarnitine |
| L-Aspartic Acid | N-Acetylaspartylglutamic acid | Acetylcholine |
| L-Glutamine | Prolylhydroxyproline | Betaine aldehyde |
| Histamine | Acetylcarnosine | Butyrylcarnitine |
| N-Acetylleucine | Homoanserine | Hydroxybutyrylcarnitine |
| Adenosine | Glutathione | Hydroxyhexanoycarnitine |
| Guanosine | Creatine | Indolelactic acid |
| Inosine | Malic acid | L-Carnitine |
| N6-Methyladenosine | 3-Oxohexadecanoic acid | L-Octanoylcarnitine |
| Adenosine monophosphate | Benzoic acid | Phenylacetic acid |
| Inosinic acid | Cinnamic acid | Phenylpyruvic acid |
| Hypoxanthine | Citric acid | 3-Indolebutyric acid |
| Xanthine | Nonanedioic acid | Hippuric acid |
| Xanthosine | Octadecanedioic acid | Hydroxypropionylcarnitine |
| 3-Methyluridine | 2-Hydroxymyristic acid | Kynurenine |
| Deoxycytidine | 3-Hydroxycapric acid | Phenylglyoxylic acid |
| Deoxyuridine | 3-Hydroxydodecanoic acid | Uric acid |
| Thymidine | Citraconic acid | Glucose 6-phosphate |
| Uridine | Citramalic acid | Ribonic acid |

Table 5S Composition of volatile flavor compounds in chicken soup (μg/mL)

| Evaluation criteria | Different salt additions/% | | | |
| --- | --- | --- | --- | --- |
| Standard/weight | 1.5 | 2.0 | 2.5 | 3.0 |
| Aldehydes | 1953.74±260.21^a^ | 2500.49±169.25^b^ | 2885.52±43.12^c^ | 2839.58±45.19^c^ |
| Alcohols | 363.87±22.54^a^ | 361.41±27.75^a^ | 366.9±20.91^a^ | 361.89±47.32^a^ |
| Furans | 35.49±19.28^b^ | 35.09±5.24^b^ | 26.29±3.68^b^ | 4.76±1.11^a^ |
| Alkene | 118.5±48.61^a^ | 114.37±32.09^a^ | 101.48±17.52^b^ | 111.87±32.75^a^ |
| Ketones | 189.52±63.32^a^ | 172.15±22.52^a^ | 209.42±4.55^a^ | 155.76±39.22^a^ |
| Esters | 6.92±0.24^b^ | 5.81±1.97^ab^ | 5.99±1.35^ab^ | 4.35±0.57^a^ |
| Aromatic | 30.31±2.79^b^ | 20.564±1.94^a^ | 24.521±1.81^a^ | 23.104±4.17^a^ |
| Others | 44.86±6.10^a^ | 90.26±25.27^b^ | 80.3±13.74^ab^ | 77.28±26.23^ab^ |

Note: *P*<0.05 is represented between different lowercase letters of the same pair, and the same letter represents *P*>0.05.
